# *Plasmodium vivax* AMA1: Implications of distinct haplotypes for immune response

**DOI:** 10.1371/journal.pntd.0008471

**Published:** 2020-07-08

**Authors:** Najara Carneiro Bittencourt, Ana Beatriz Iung Enembreck da Silva, Natália Silveira Virgili, Ana Paula Schappo, João Henrique D. B. Gervásio, Tamirys S. Pimenta, Mario A. Kujbida Junior, Ana Maria R. S. Ventura, Rosana M. F. Libonati, João Luiz Silva-Filho, Hellen Geremias dos Santos, Stefanie C. P. Lopes, Marcus V. G. Lacerda, Ricardo L. D. Machado, Fabio T. M. Costa, Letusa Albrecht

**Affiliations:** 1 Departamento de Genética, Evolução, Microbiologia e Imunologia, Instituto de Biologia, Universidade Estadual de Campinas (UNICAMP), Campinas, SP, Brazil; 2 Instituto Carlos Chagas, Fundação Oswaldo Cruz–FIOCRUZ. Curitiba, PR, Brazil; 3 Núcleo de Medicina Tropical, Universidade Federal do Pará, Belém, PA, Brazil; 4 Fundação de Medicina Tropical Dr. Heitor Vieira Dourado, Gerência de Malária, Manaus, AM, Brazil; 5 Instituto Leônidas & Maria Deane, FIOCRUZ-AMAZONAS, Manaus, AM, Brazil; 6 Centro de Investigação de Microrganismos, Universidade Federal Fluminense, RJ, Brazil; Johns Hopkins Bloomberg School of Public Health, UNITED STATES

## Abstract

In Brazil, *Plasmodium vivax* infection accounts for around 80% of malaria cases. This infection has a substantial impact on the productivity of the local population as the course of the disease is usually prolonged and the development of acquired immunity in endemic areas takes several years. The recent emergence of drug-resistant strains has intensified research on alternative control methods such as vaccines. There is currently no effective available vaccine against malaria; however, numerous candidates have been studied in the past several years. One of the leading candidates is apical membrane antigen 1 (AMA1). This protein is involved in the invasion of Apicomplexa parasites into host cells, participating in the formation of a moving junction. Understanding how the genetic diversity of an antigen influences the immune response is highly important for vaccine development. In this study, we analyzed the diversity of AMA1 from Brazilian *P*. *vivax* isolates and 19 haplotypes of *P*. *vivax* were found. Among those sequences, 33 nonsynonymous PvAMA1 amino acid sites were identified, whereas 20 of these sites were determined to be located in predicted B-cell epitopes. Nonsynonymous mutations were evaluated for their influence on the immune recognition of these antigens. Two distinct haplotypes, 5 and 16, were expressed and evaluated for reactivity in individuals from northern Brazil. Both PvAMA1 variants were reactive. Moreover, the IgG antibody response to these two PvAMA1 variants was analyzed in an exposed but noninfected population from a *P*. *vivax* endemic area. Interestingly, over 40% of this population had antibodies recognizing both variants. These results have implications for the design of a vaccine based on a polymorphic antigen.

## Author summary

*Plasmodium vivax* is the most abundant *Plasmodium* species in Brazil. While this species has been neglected for many years, the recent emergence of drug-resistant strains and the absence of a vaccine intensified the efforts for a better control method. Naturally acquired immune response analysis is a useful tool for understanding the antigenicity of *Plasmodium* proteins and evaluating the potential of a vaccine candidate. In this study, the genetic variability of one of the leading *P*. *vivax* vaccine candidates (PvAMA1) was analyzed. Two distinct variants were expressed and the antibody response was evaluated in infected and noninfected individuals in the Brazilian Amazon. This improved understanding of the magnitude and dynamics of the antibody response will contribute to the knowledge of a vaccine candidate and open new perspectives in vivax malaria vaccine development.

## Introduction

Malaria remains one of the greatest global public health problems, with approximately 3.3 billion people being at risk of infection. In South and Central America, *P*. *vivax* accounts for over 70% of malaria cases, thereby representing the most prevalent *Plasmodium* species. In Brazil, approximately 174,000 cases of vivax malaria were reported last year, which corresponds to 89.2% of the total number of malaria cases [1, 2].

*P*. *vivax* infection can be treated with chemotherapy; however, resistance is rising and alternative therapies are increasingly desirable [3]. No vaccines against vivax malaria are available to date. Nevertheless, several vaccine candidates have been studied [4, 5]. Among these candidates, the leading antigen candidate for vivax malaria is apical membrane antigen 1 (AMA1).

AMA1 is expressed in the microneme of Apicomplexa parasites, present in all *Plasmodium* species. AMA1 is involved in the process of parasite invasion into host cells [6, 7], and, working together with proteins of the rhoptry neck protein (RON) complex, in the formation of the moving junction (MJ) [8]. Moreover, AMA1 is also involved in the invasion of sporozoites into human hepatocytes [9]. This antigen presents a unique opportunity as a multi-stage vaccine target. Attempts to silence *ama1* of *P*. *falciparum* and *Toxoplasma gondii* have shown that AMA1 has a central role in merozoite invasion, indicating that this protein might be essential to parasite survival [6, 10]. Studies with *P*. *berghei* sporozoites showed that without AMA1, parasites can invade and develop in hepatocytes, but subsequently formed merozoites cannot invade erythrocytes. These results suggested that AMA1 has a fundamental role in the blood stage cycle, which could be involved in the connection, redirection and stabilization of erythrocyte binding [11]. Taking into account the vital importance of AMA1 to the parasite, this protein has also been considered an important target for parasite control [12, 13].

The immunogenic potential of AMA1 was first observed after immunization of *Rhesus* monkeys with the native protein purified from *P*. *knowlesi*, which induced significant protection against infection [14]. Furthermore, immunization of mice or monkeys with recombinant protein derived from *P*. *chabaudi* or *P*. *fragile*, respectively, was able to induce significant protection [15, 16]. In addition, immune epidemiological studies conducted in Brazil showed that *P*. *vivax* AMA1 is highly immunogenic in natural infections [17].

AMA1 contains four regions: 1) pro-sequence, 2) rich cysteine ectodomain, 3) transmembrane domain and 4) cytoplasmic region. Within the ectodomain, the disulfide bridges formed by cysteine residues enable them to be separated into three domains (D), referred to as DI, DII, and DIII [18]. The highest rate of mutations and genetic diversity has been shown within DI [19]. DII has a high degree of amino acid conservation and is the most immunogenic region of AMA1 in *P*. *falciparum* and *P*. *vivax* [20], being recognized for human antibodies after natural infection [17]. Although the genetic diversity of malaria antigens has been extensively studied, this subject is a matter of high importance, since the effectiveness of a vaccine or a drug might depend on the conservation of specific amino acid sites.

This study examined the impact of the genetic diversity of *P*. vivax AMA1 (PvAMA1) on the reactive profile of two distinct haplotypes in an endemic malaria region. The hypothesis is that genetic polymorphisms might direct the immune response.

## Methods

### Ethics statements

Ethical permission for the study was obtained by the local ethical committee in Manaus, AM, Brazil (CAAE-0044.0.114.000–11) and Itaituba, PA, (Research Ethics Committee of the Universidade Federal do Pará (Protocol no. 1.088.855/2015) and of Instituto Evandro Chagas (Protocol no. 1.219.346/2015). All samples were collected after obtaining written consent from all individuals.

### Sample collection

Blood samples for DNA extraction and sequence analysis were collected from patients with malaria who were seeking medical care at *Fundação de Medicina Tropical Dr*. *Heitor Vieira Dourado*, Manaus, Amazonas state, a low transmission region located in northern Brazil from 2011 to 2012. Blood samples were collected using BD Vacutainer with sodium citrate anticoagulant. Blood analysis was carried out just after blood collection using a Sysmex KX21N (Sysmex Corporation-Roche, Japan).

A thin blood smear was prepared from each blood sample to determine species of malaria parasites. Any mixed *Plasmodium* spp. infections were detected and excluded from analysis after a PCR species-specific diagnostic was performed as previously described [21].

Plasma samples were collected in Manaus (2011–2012), Amazonas-Brazil (-03^o^06’26”S; 60^0^01’34”W) and in Itaituba, Pará, Brazil (04°16'34”S; 55°59'01''W), a gold mining area, located in the most southwestern part of the state of Pará. Plasma from Itaituba were collected during the year of 2016. Samples from Manaus were from non-severe patients currently infected with *P*. *vivax* (n = 33). *P*. *vivax* samples from Itaituba were from two distinct groups: 1) non-severe patients acutely infected with *P*. *vivax* (n = 79), 2) individuals who were previously, but not currently, infected with malaria (n = 92). After collection of peripheral blood, patients received treatment following national guidelines.

The annual parasite incidence designates Itaituba as an area with a high risk of malaria transmission (102.0 cases/1000 inhabitants per year). In contrast, Manaus presents low malaria transmission (5.8 cases/1000 inhabitants per year). In northern Brazil, the predominant species is *P*. *vivax*, although there are some registered cases of *P*. *falciparum* and *P*. *malariae* [22].

### Genomic DNA extraction, amplification and sequencing of *pvama1*

Genomic DNA from malaria infected peripheral blood was extracted using the phenol:chloroform method [23]. Four overlapping fragments of PvAMA1 were amplified as previously described [24]. To minimize nucleotide mis-incorporation, all PCR reactions were performed using Platinum Taq High Fidelity (Thermo Fisher Scientific). Each PCR product was analyzed by electrophoresis in a 1.2% agarose gel. PCR products were purified using the QIAquick PCR Purification Kit (Qiagen GmbH, Hilden, Germany). The purified PCR products were stored at -20°C until sequencing was performed. PCR fragments were sequenced in forward and reverse directions using the BigDye Terminator Cycle Sequencing Kit 3.1 (PerkinElmer, MA, USA) at the automatic sequencer ABI 3100 (Applied Biosystems, CA, USA) at the LACTAD platform at UNICAMP (Campinas, SP, Brazil).

### Genetic analysis of *pvama1*

Sequences of *pvama1* from 40 isolates from Manaus were analyzed. Fragments of each sample were analyzed and assembled using the software *Phred* [25] and *pregap4*, present in the *Staden Package* [26]. Sequence data were deposited in GenBank (Accession numbers: GenBank MH049550 to MH049589). The coding gene sequence, measuring 1,689 bp in length, from *pvama1* (nt1–1,689 and aa1–562) was analyzed using MEGA 7.026 software. These sequences were aligned together with the reference sequence PvAMA1 from the *P*. *vivax* Sal-I strain (PVX_092275) to identify single nucleotide polymorphisms (SNPs) [27].

Each of the 40 sequences was parsed in the 3 functional domains of the protein, which were analyzed as its own individual set of data. The DNA polymorphism analysis tool (DnaSP 6.12.03) was used to calculate the number of polymorphisms, number of haplotypes, gene diversity and nucleotide diversity (π). Tajima's D test was performed to assess the selective pressure on the *pvama1* gene using the same software. The frequency of polymorphic sites in DI, DII and DII was compared through Chi-square test. Public sequences from previous studies [20, 24, 28–35] were also compared to sequences from Manaus.

The values of synonymous substitution (dS) and nonsynonymous substitution (dN) were obtained by computing the overall mean distance of the sequences pairwise with the substitution type syn-nonsynonymous, using Nei-Gojobori [36] with Jukes and Cantor correction [37] as implemented in the MEGA 7.026 software. To obtain dS values, only synonymous substitutions were accounted for followed by calculating the mean in each sequence. The same method was used to obtain dN values, taking into consideration only nonsynonymous substitutions. Standard errors of dS and dN were estimated by bootstrap with 1000 replications. To determine whether natural selection contributes to diversity of PvAMA1, the ratio of nonsynonymous to synonymous substitutions (dN/dS) was evaluated for the entire protein as well as for DI, DII and DIII through Z-test using MEGA 7.026 software.

To obtain a dendrogram showing genetic proximity, the online program PhyML 3.0 (http://www.atgc-montpellier.fr/phyml/) was used. The Bayesian information criterion (BIC) was applied to select a final model with 1000 bootstrap resampling.

### Predicted linear B-cell epitopes and position of mutations on the 3D protein structure

B-cell epitope predictions for PvAMA1 from the reference *P*. *vivax* genome Sal1 (PVX_092275) were conducted at BepiPred-2.0: Sequential B-Cell Epitope Predictor [38]. An epitope threshold of 0.5 with a length of at least 5 amino acids long was applied. B-cell epitopes and mutation sites inside predicted B cell epitopes were visualized as a three-dimensional structure of PvAMA1 (PDB ID: 1W81) using Pymol [39].

### Recombinant PvAMA1 (rPvAMA1) expression and purification

Sequences corresponding to the PvAMA1 ectodomain (nucleotides 130 to 1490) from two PvAMA1 haplotypes (H5 and H16) were cloned into the pGEX 4T-1 vector and transformed into *E*. *coli* Arctic Express (DE3) competent cells (Agilent Technologies) for protein expression.

Bacteria were cultured according to the manufacturer’s instructions. Briefly, 100 mL of overnight culture was transferred into 3000 mL of LB containing gentamicin (20 μg/mL) and ampicillin (100 μg/mL). The culture was placed at 37°C and grown until reaching an optical density (OD)_600_ of 0.6. Cells were induced by the addition of 0.08 mM IPTG for 16 h at 12°C. The culture was collected by centrifugation (6.000 g, 10 min), and resuspended in 20 mL of lysis buffer (10 mM Tris-HCl pH 8, 150 mM NaCl, 1 mM EDTA) in the presence of 1X Complete Protease Inhibitor Cocktail (Roche, Mannheim, Germany) and incubated for 1 h on ice. The culture was then disrupted using an M-110L Pneumatic High Shear Fluid Processor (Microfluidcs). Cell fragments were pelleted by 30 min of centrifugation at 10000 x g. The supernatant was collected and tested by SDS-PAGE and western blotting to confirm the recombinant protein expression. The synthesized recombinant proteins were purified using Glutathione Sepharose 4B (GE Heatlhcare) according to the manufacturer’s instructions.

### Circular Dichroism Analysis

CD (Circular dichroism) spectroscopy was performed to predict the secondary structure of the two PvAMA1 variants. CD spectrum was recorded on a JASCO J-815 CD spectrometer. PvAMA1V5 and PvAMA1V16 proteins were buffer exchanged into 15mM Potassium phosphate pH 8.0. and concentrated to 0.16 mg/mL. Proteins were scanned from 260 to 190 nm using a 0.1 cm path length cuvette. Data were corrected for the baseline with respect to buffer and analyzed using Spectra manager version 2. The ellipticity (θ) values were calculated according to Kelly et al., 2005 [40].

### Measurement of naturally acquired antibody to rPvAMA1 variants

Naturally acquired IgG antibodies against two rPvAMA1 variants were measured in plasma samples by direct enzyme-linked immunosorbent assay (ELISA). First, plasma samples from infected patients from Manaus were evaluated. Subsequently, samples collected in gold-mining regions in Itaituba from infected and noninfected individuals who had previously been exposed to vivax malaria were analyzed to evaluate the persistence of antibodies against the PvAMA1 variants.

Briefly, high binding plates were coated with 50 μl of each rPvAMA1 variant (PvAMA1V5 and PvAMA1V16) at 5 μg/mL in coating buffer (0.05M carbonate-bicarbonate pH 9.6), overnight, at room temperature. Plasma samples diluted 1:100 were added to each well and incubated for 1 h at room temperature. Goat anti-human IgG conjugated to peroxidase (Sigma-Aldrich) was used for detection at a dilution of 1:2000. The presence of IgG antibodies was detected by O-phenylenediamine diluted in phosphate-citrate buffer containing 0.003% hydrogen peroxide. The OD was measured at 490 nm using CLARIOstar data analysis. All OD_490_ values were normalized using the values of the anti-GST control. Plasma samples of 20 healthy individuals from a non-endemic area were used as negative controls. The cutoff value was calculated as the mean plus three standard deviations of the negative control. The reactivity indexes were obtained from the ratio of absorbance values of each sample and the cut off value. IgG reactive indexes greater than one were considered to be positive. Bias produced by a possible reactivity of the GST tag was evaluated as described by Bittencourt et al., 2018 [41].

### Quantification of Plasmatic Cytokines

The plasma cytokines IL-6, IFN-γ, IL-10 and TNF-α were quantified by flow cytometry using IL-6, IL-10, IFN-γ, TNF-α Human Flex Set (BD Bioscience Pharmingen, San Diego, CA, USA) following the manufacturer’s instructions. The mean fluorescence intensity of each bead cluster was determined, and the forth logistic regression was applied to build the standard curve. Data analyses were performed using the FACSDiva software (BD Biosciences, San Jose, CA, USA).

### Statistical analysis

Spearman’s correlation coefficient (*rho*) was computed to examine the relationship between PvAMA1V5 and PvAMA1V16 reactivity indexes and between these variables and age, parasitemia, platelets, total lymphocytes, number of red blood cells, hematocrit and hemoglobin. To assess if *rho* was statistically different from zero, we applied t*-*test. Additionally, paired comparison between PvAMA1V5 and PvAMA1V16 reactivity indexes was evaluated through Wilcoxon signed-rank test. A significance level of 0.05 for all statistical tests were applied.

## Results

### Diversity of PvAMA1 in Brazil

A total of 40 sequences of *pvama1* from *P*. *vivax* isolates from Manaus were analyzed from nucleotides 1 to 1689, which corresponds to the *pvama1* full length coding sequence with *P*. *vivax* Sal-1(PVX_092275) as a reference sequence.

Among the 1689 nucleotides analyzed 51 SNPs were found. The overall nucleotide diversity was π = 0.0062 (Table 1). The highest pairwise diversity (π) was found at DI (π = 0.0111) followed by DII (π = 0.0055) and DIII (π = 0.0030) (Table 1). Tajima's D value was -0.4721. The entire protein and DI had a dN/dS ratio greater than 1, however, no significant differences were found (Table 1). When sequences from Manaus were compared with publish sequences from other places in the world [20, 24, 28–35], the overall nucleotide diversity was π = 0.00925 (Table 2).

**Table 1:** Comparison of *pvama1* genetic diversity among isolates from Brazil.

Region	Residues	S	H	Gene diversity (sd)	π (Tajima D)	dN (s.e)	dS (s.e)	dN/dS	p-value
Entire gene	1–562	51	19	0.923(0.026)	0.0062(-0.4721)	0.0066 (0.0015)	0.0047 (0.0018)	1,40	0.27
DI	42–248	27	10	0.719(0.066)	0.0110 (0.2816)	0.0124 (0.0036)	0.0069 (0.0041)	1,79	0.125
DII	249–385	14	10	0.868(0.024)	0.0055 (-0.9898)	0.0052 (0.0022)	0.0069 (0.0047)	0,76	0.69
DIII	386–487	4	4	0.683(0.031)	0.0030 (-0,0407)	0.0037 (0.0023)	0 (0)	-	0.11

S = number of polymorphisms; H = number of haplotypes, sd = standard deviation; π = nucleotide diversity; dN = nonsynonymous substitution; dS = synonymous substitution.

**Table 2:** Comparison of *pvama1* genetic diversity among isolates from worldwide.

Region	Number of sequences	Number of polymorphic sites	Number of haplotypes	π(Tajima's D)	Gene diversity (sd)
**World**	283	85	172	0.00925(-0.18579)	0.9905(0.0017)
**Americas**	97	45	34	0.00749(0.65952)	0.946(0.01)
Brazil*	145	27	28	0.01684(0,60764)	0.880(0.016)

Only sequences larger than 1400 bp from the World and the Americas we considered. *Brazilian partial sequences were analyzed (PvAMA1- domain I).

**Fig 1:**
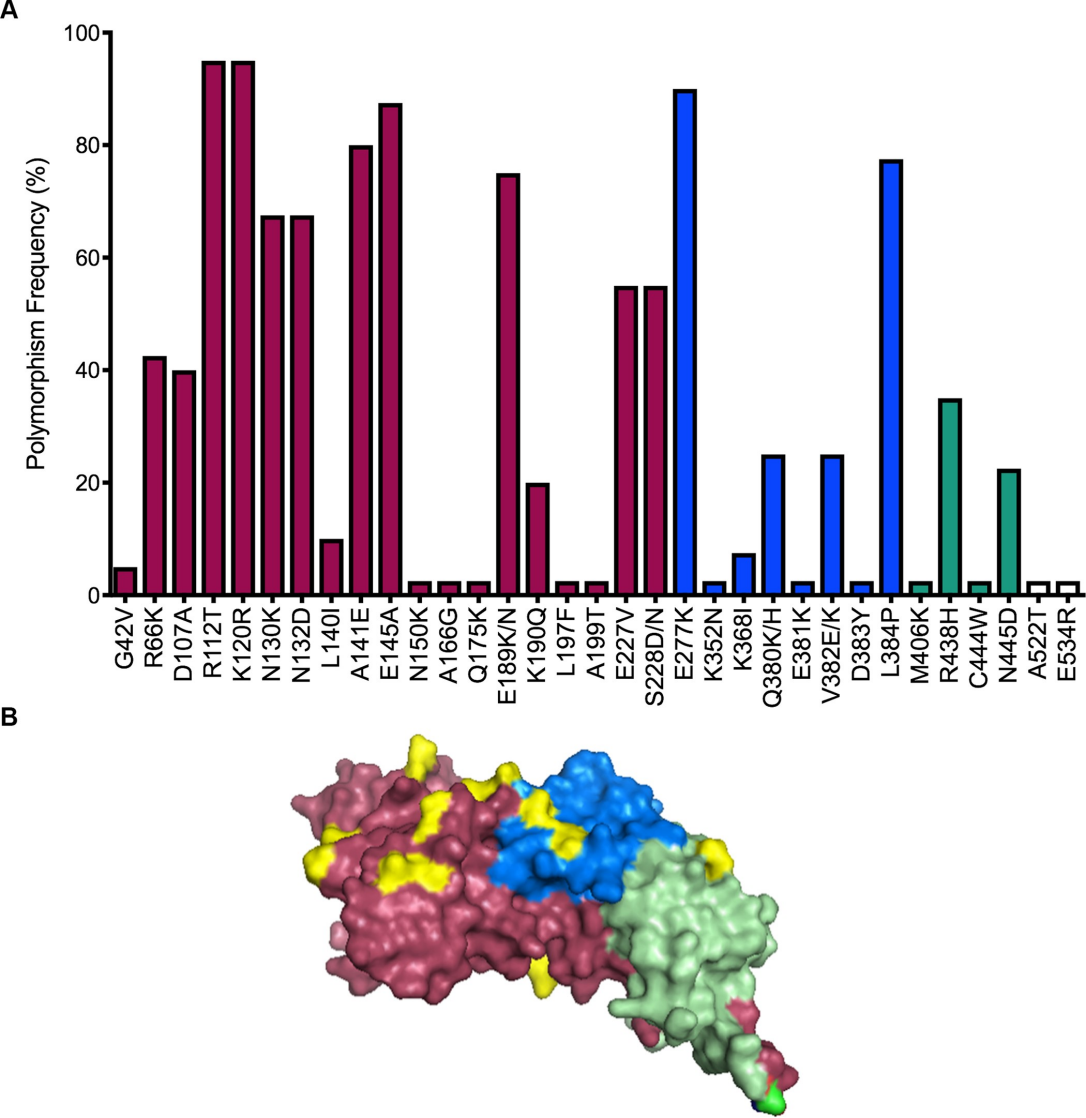
Frequency and location of polymorphic sites found at PvAMA1 from Brazilian isolates. **A.** The frequencies of polymorphic sites are represented in dark pink, blue and green bars located in DI, DII, DIII, respectively, corresponding to the ectodomain. White bars indicate amino acid changes outside the ectodomain. **B.** Polymorphic sites in the 3D model of the PvAMA1 ectodomain based on the crystal structure (PDB ID: 1W81). Polymorphic amino acid residues on the PvAMA1 structure located at haplotypes 5 and 16 are colored yellow. Domain I, II and III are colored in dark pink, blue and light green, respectively.

Thirty-three polymorphic sites were found at the amino acid level with 12 of them being singleton sites. Twenty-nine dimorphic sites and four trimorphic sites were found (E189K/N, S228D/N, Q380K/H and V382E/K) across the PvAMA1 sequence (Fig 1A). Within the ectodomain of PvAMA1 we found 30 polymorphic sites. The majority of polymorphisms were located at DI, where 18 polymorphic sites were present followed by 8 sites at DII and 4 sites at DIII. Although DI had the highest number of polymorphic amino acids, there was no significant difference between the frequency of polymorphic sites among the domains (Chi-square test, *p* = 0.356).

### PvAMA1 haplotype frequency in Brazilian field isolates

Among the 40 isolates analyzed, 19 distinct haplotypes were found with haplotype diversity (Hd) of 0.923 (Table 1). The frequencies of each haplotype are shown in Table 3. The most frequent haplotype was H5 (22.5%). It is worth noting that 12 haplotypes were unique, with a frequency of 2.5% (Table 3). When sequences from Manaus were analyzed together with 243 published sequences from distinct geographical regions, 173 haplotypes were found. However, the most frequent haplotype found in Brazil (H5) was not found anywhere else. Published PvAMA1 sequences from Brazil are limited to DI. When this domain was evaluated 28 haplotypes were found. Sequences corresponding H5 was found also in a previous study from Brazil [34] (Table 2).

**Table 3:** Polymorphic sites across each haplotype and the presence of predicted linear B-cell epitopes on variant PvAMA1 sites.

H/AA	H(%)	Amino acid residue (position)																																
Pv_SalI		G (42)	R (66)	D (107)	R (112)	K (120)	N (130)	N (132)	L (140)	A (141)	E (145)	N (150)	A (166)	Q (175)	E (189)	K (190)	L (197)	A (199)	E (227)	S (228)	E (277)	K (352)	K (368)	Q (380)	E (381)	V (382)	D (383)	L (384)	M (406)	R (438)	C (444)	N (445)	A (522)	E (534)
**H1**	7,50	.	.	.	T	R	K	D	.	E	A	.	.	.	N	.	.	.	V	D	K	.	.	.	.	.	.	.	.	.	.	D	.	.
**H2**	2,50	.	.	.	T	R	K	D	.	E	A	.	.	K	N	.	.	.	V	D	K	.	.	.	.	.	.	.	.	.	.	D	.	.
**H3**	10,00	.	.	.	T	R	K	D	.	E	A	.	.	.	N	.	.	.	V	D	.	.	.	.	.	.	.	.	.	.	.	.	.	.
**H4**	2,50	.	.	.	T	R	K	D	.	E	A	.	.	.	N	.	.	.	V	D	K	.	.	.	.	.	.	.	K	.	W	.	.	.
**H5**	**22,50**	.	.	.	**T**	**R**	**K**	**D**	.	**E**	**A**	.	.	.	**N**	.	.	.	**V**	**D**	**K**	.	.	.	.	**E**	.	**P**	.	.	.	.	.	.
**H6**	5,00	.	.	.	T	R	K	D	.	E	A	.	.	.	N	.	.	.	V	D	K	.	.	.	.	.	.	P	.	H	.	.	.	.
**H7**	2,50	.	.	.	T	R	K	D	.	E	A	.	.	.	N	.	.	.	V	D	K	.	.	.	.	.	.	P	.	H	.	.	.	.
**H8**	2,50	.	K	.	T	R	K	D	.	E	A	.	.	.	K	.	.	.	V	N	K	.	.	K	.	.	.	P	.	H	.	.	.	.
**H9**	7,50	.	K	A	T	R	K	D	.	.	.	.	.	.	.	.	.	.	.	.	K	.	.	.	.	.	.	P	.	.	.	D	.	.
**H10**	2,50	.	K	A	T	R	K	D	.	.	.	.	.	.	.	.	.	.	.	.	K	.	.	H	K	K	Y	P	.	.	.	D	.	.
**H11**	2,50	.	K	A	T	R	K	D	.	E	.	.	.	.	.	.	.	.	.	.	K	.	.	.	.	.	.	P	.	H	.	.	.	.
**H12**	12,50	.	K	A	T	R	.	.	.	E	A	.	.	.	K	Q	.	.	.	.	K	.	.	K	.	.	.	P	.	H	.	.	.	.
**H13**	2,50	.	K	A	T	R	.	.	.	E	A	.	.	.	K	Q	.	.	.	.	K	.	.	K	.	.	.	P	.	H	.	.	.	R
**H14**	2,50	.	K	A	T	R	.	.	.	E	A	K	G	.	K	Q	F	T	.	.	K	.	.	K	.	.	.	P	.	H	.	.	T	.
**H15**	2,50	.	K	A	T	R	.	.	I	.	A	.	.	.	.	.	.	.	.	.	K	.	.	.	.	.	.	P	.	H	.	.	.	.
**H16**	**2,50**	.	**K**	**A**	**T**	**R**	.	.	**I**	.	**A**	.	.	.	.	.	.	.	.	.	**K**	.	.	**K**	.	.	.	**P**	.	**H**	.	.	.	.
**H17**	2,50	.	K	A	T	R	.	.	.	E	A	.	.	.	K	Q	.	.	.	.	K	N	I	.	.	.	.	P	.	.	.	.	.	.
**H18**	2,50	.	K	A	T	R	.	.	.	E	A	.	.	.	.	.	.	.	.	.	K	.	.	.	.	.	.	P	.	.	.	D	.	.
**H19**	5,00	V	.	.	.	.	.	.	I	.	A	.	.	.	.	.	.	.	.	.	K	.	I	.	.	.	.	P	.	.	.	.	.	.
									**DI**	** **	** **	** **	** **	** **	** **	** **	** **	** **	** **	** **	** **	** **	** **	** **	**DII**	** **	** **	** **	** **	** **	**DIII**	** **		

H = n° of haplotypes; AA = amino acid; DI = domain (pink) I; DII = domain II (dark blue); DIII = domain III (dark green). Amino acids position colored in light blue indicate predicted B cell epitopes. Orange and gray amino acids represent mutation points and conserved positions between H5 and H16 sequences respectively.

All haplotypes detected were genetically distributed in a dendrogram (S1 Fig). Two of these haplotypes were chosen to analyze the reactivity of those variants in samples from individuals from malaria-endemic regions: one haplotype with high frequency (H5 = 22.5%) and another haplotype with low frequency (H16 = 2.5%).

### Several mutation points are located at B-cell epitopes

Predicted B-cell epitopes are listed in S1 Table. Twenty-two predicted linear B-cell epitopes were found across PvAMA1, with 10 containing at least one polymorphic site. Among the 33 polymorphic sites found at PvAMA1, 20 sites are located at predicted B-cell epitopes and 12 amino acid changes are located in H5 and H16 as shown in the 3D model in Fig 1B.

Haplotypes 5 and 16 differed in 12 polymorphic sites across the ectodomain. Nine polymorphic sites were located in DI, two sites in DII and only one in DIII. Among those sites, seven sites are located in predicted B-cell epitopes (S1 Table, Fig 1B).

### Antibody responses differ depending on haplotype

Recombinant proteins were characterized in terms of purity and quality by SDS-PAGE and structure by CD analysis (S2 Fig). The CD spectra suggest a higher presence of α-helical characterized by two negative bands of similar magnitude, and a positive band at ~ 190 nm [42]. The same global secondary structure pattern was visualized when PvAMA1V5 and PvAMA1V16 proteins were compared.

**Fig 2:**
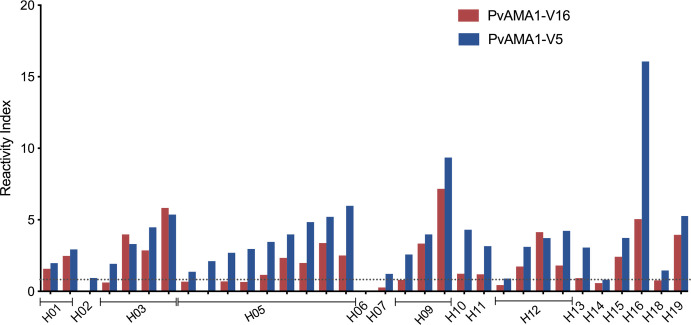
Antibody response against variants 5 and 16 of individuals from Manaus infected with distinct PvAMA1 variants. Each bar represents the reactivity index of a particular patient infected by a given haplotype. Colors blue and red indicate IgG antibodies against PvAMA1V5 and PvAMA1V16 respectively. Reactivity indexes higher than 1 are positive and visualized above the dotted line.

To understand the importance of sequence variability, IgG responses towards both variants were evaluated in a subset of the population previously characterized for genetic diversity. Most of the population from Manaus (87.9%) had antibodies against PvAMA1V5 and 60.6% had antibodies towards PvAMA1V16. All patients who had antibodies to PvAMA1V16 also had antibodies to PvAMA1V5 (Fig 2).

**Fig 3:**
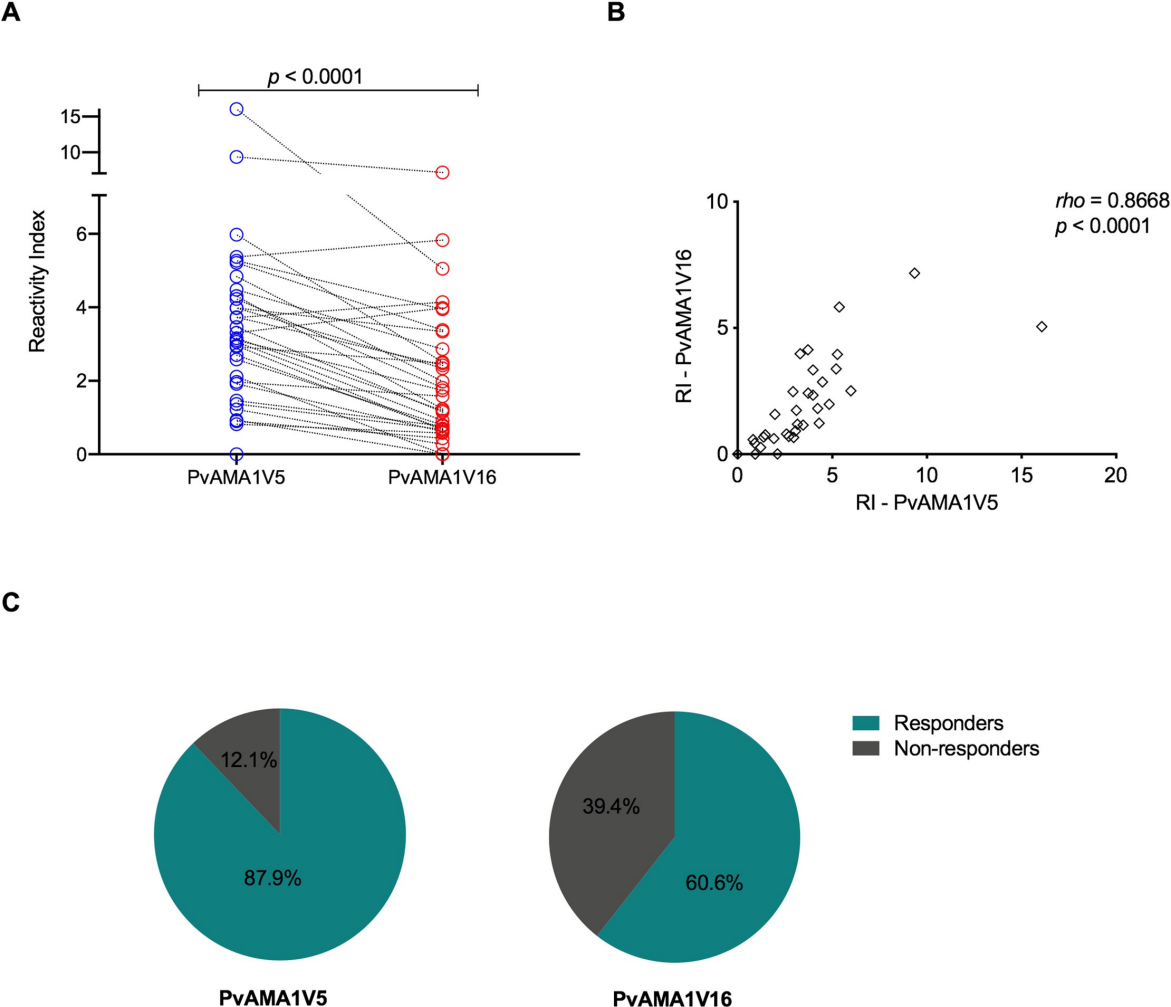
Correlation and paired comparison of antibody responses to PvAMA1 variants from Manaus. Reactivity indexes of A) Paired PvAMA1V5 and PvAMA1V16 IgG responses comparison (Wilcoxon signed-rank test, p<0.0001). B) Spearman correlation coefficient (*rho*) between PvAMA1V5 and PvAMA1V16 reactivity indexes and t-test p-value (*p*), C) Prevalence of responders to PvAMA1V5 and PvAMA1V16.

All samples that contained haplotype PvAMA1H5 (n = 9) also had antibodies recognizing this sequence (PvAMA1V5). Nevertheless, only 5 out of 9 of individuals containing PvAMA1H5 had antibodies against PvAMA1V16 (Fig 2). On the other hand, the individual infected with PvAMA1H16 parasites had antibodies to both variants, however with higher reactivity against PvAMA1V5 in most cases (*p<* 0.001 for Wilcoxon signed-rank test, Fig 3A). The antibody response against both variants was strongly correlated (Spearman test, *rho =* 0.867 and *p*< 0.0001) (Fig 3B).

Although the sample size was notably limited, individuals infected with parasites belonging to haplotypes H2, H6 and H14 had no antibodies to either of the variants analyzed. Individuals infected with H7, H13 and H18 did not have antibodies to PvAMA1V16. On the other hand, individuals infected with parasites containing H1, H10, H11, H15, H16 and H19 had antibodies towards PvAMA1V5 as well as PvAMA1V16.

**Fig 4:**
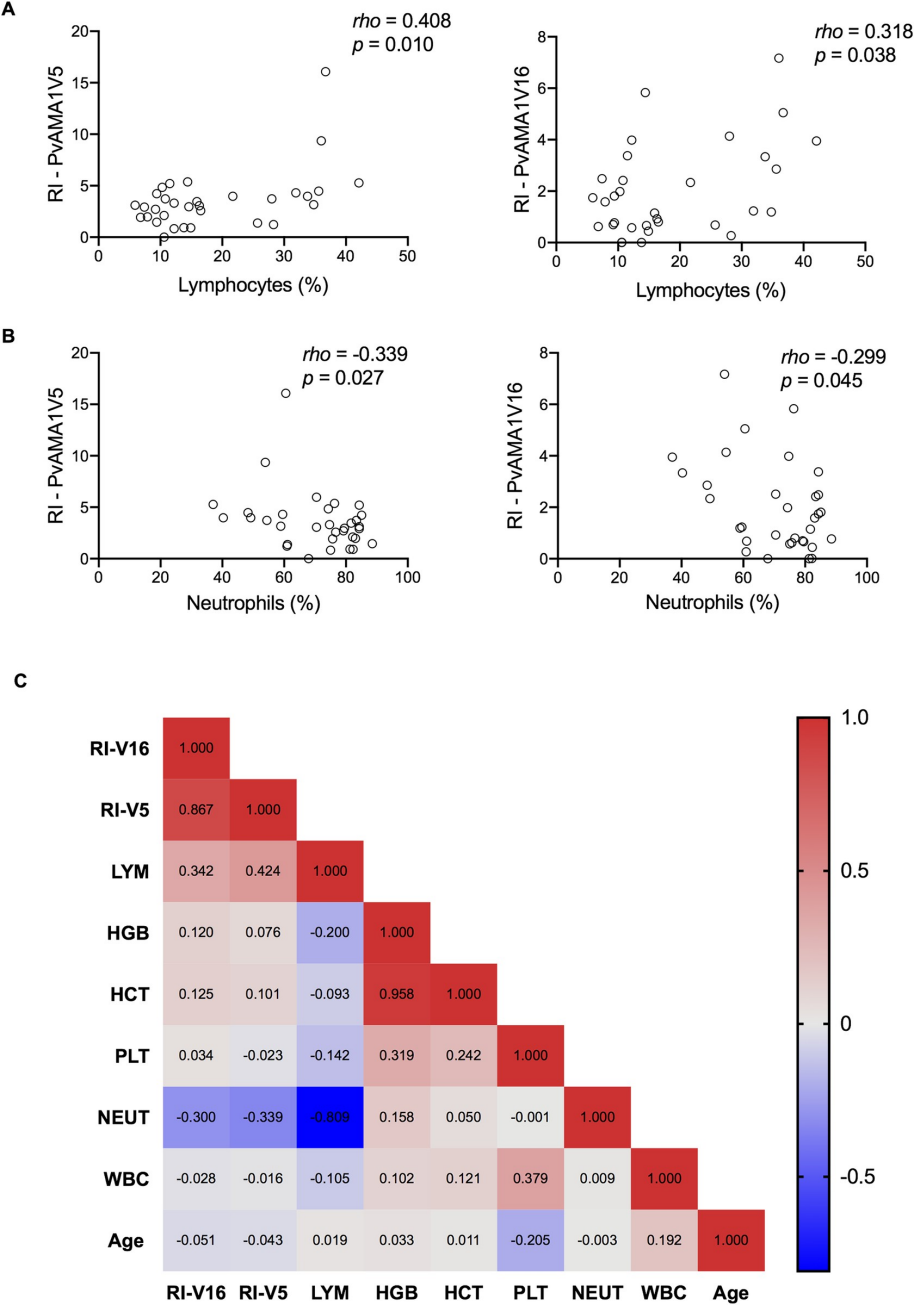
Correlation between hematological parameters and reactivity indexes of individuals from Manaus. A) Spearman correlation coefficient (*rho*) and t*-*test p-value (*p*) between reactivity indexes and lymphocytes frequency, B) *rho* and *p* between reactivity indexes and neutrophils and C) Spearman correlation matrix of hematological parameters, age and reactivity indexes. Scale is based on colors from blue (perfect positive correlation, 1) to red (perfect negative correlation, -1). Each line represents one variable.

The correlation of antibody response with hematological parameters and age was also analyzed. The number of lymphocytes presented a moderate positive correlation with the antibody response against PvAMA1V5 (Fig 4A, *rho =* 0.408 and *p =* 0.010) and PvAMA1V16 (Fig 4A, *rho =* 0.318 and *p =* 0.038). While a moderate negative correlation was observed between the immune reactivity and neutrophils (S3B Fig, PvAMA1V5 *rho =* -0.339 *p =* 0.027 and PvAMA1V16 *rho =* -0.299 *p =* 0.045). However, no other association was found when hematocrit, platelets, total number of white blood cells or age were analyzed (Fig 4C).

### Antibody responses to PvAMA1 variants are maintained in the absence of *P*. *vivax* infection

The importance of these polymorphisms for antibody response was also evaluated in a geographically distinct population in Itaituba, state of Pará. Among the 171 individuals evaluated, 70.8% (n = 121) had antibodies to PvAMA1V5 and 50.3% (n = 86) had antibodies to PvAMA1V16 (S3 Fig). The higher reactivity index was against PvAMA1V5 for most individuals (*p <* 0.0001 for Wilcoxon signed-rank test), 81/171 (47.4%) individuals had antibodies to both variants while 45/171 (26.3%) of the studied population had no antibodies to these variants.

**Fig 5:**
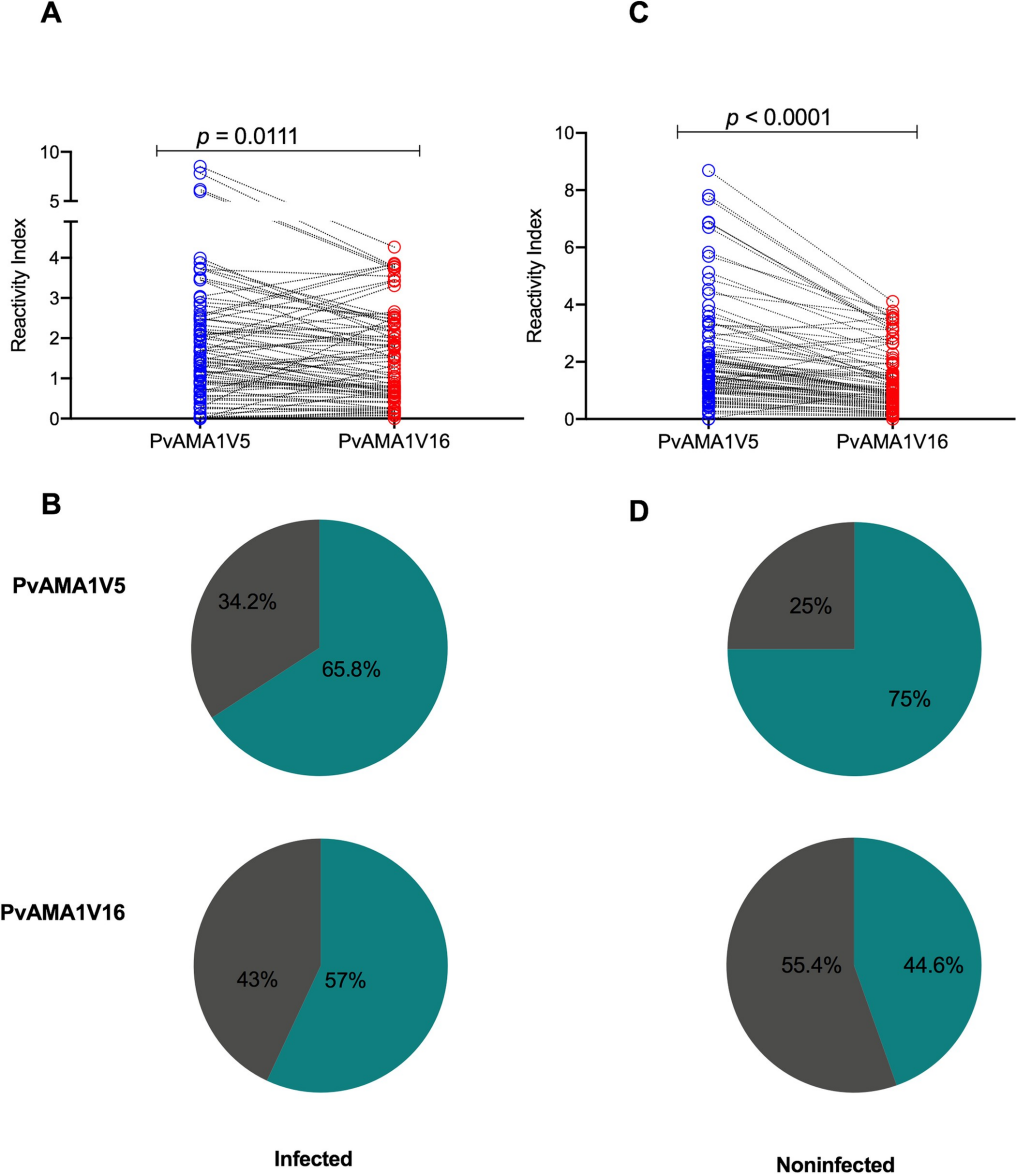
Paired comparison of antibody response to PvAMA1 variants in infected and noninfected individuals from Itaituba. A) Paired PvAMA1V5 and PvAMA1V16 IgG responses comparison in infected individuals (Wilcoxon signed-rank test p-value). B) Prevalence of responders towards both variants in infected group. C) PvAMA1V5 and PvAMA1V16 IgG responses comparison in noninfected individuals (Wilcoxon signed-rank test p-value). D) Prevalence of immune responses in noninfected individuals.

In the infected group, 51.9% (n = 41) had antibodies to both variants while 29.1% (n = 23) had no antibodies to either variant. In this population, 65.8% (n = 52) of individuals had an immune response against PvAMA1V5 and 57% (n = 45) against PvAMA1V16. In the noninfected population, 43.5% (n = 40) had antibodies to both variants, and 23.9% (n = 22) of individuals did not have antibodies to either variant. In this group, 75% (n = 69) had antibodies against PvAMA1V5 while 44.6% (n = 41) had antibodies against PvAMA1V16 (Fig 5). Similar to individuals from Manaus, in this population, there was a strong correlation between the antibody response towards both variants (S3B, S4A and S4C Figs).

In analysis including all individuals from Itaituba sample, PvAMA1V5 antibody response presented a weak but statistically significant positive correlation with age (*rho* = 0.249 and *p* = 0.001); for PvAMA1V16 antibody response, this correlation was lower and with no statistical significance at 0.05 level (*rho* = 0.117 and *p* = 0.064). When the analyses were performed according to infected and noninfected groups, similar results were observed, with statistical significance only for correlation between PvAMA1V5 antibody response and age for the latter group (*rho* = 0.2913 and *p* = 0.0024) (S5 Fig). No correlation was found between the plasma cytokine level and the PvAMA1 antibody response (S6 Fig).

## Discussion

AMA1 plays an essential role in erythrocyte invasion and is one of the major potential vaccine candidates against malaria. This protein is exposed to the immune system and is capable of inducing an antibody response that blocks parasite invasion into host cells [43, 44]. A major challenge in the development of an efficient malaria vaccine is the presence of high levels of polymorphisms in the parasite antigens that are under immune pressure. Strain-specific immunity could enable parasites to escape vaccine-induced antibodies [45]. In this study, polymorphisms in the PvAMA1 antigen from Brazilian *P*. *vivax* isolates were identified and the influence of two different PvAMA1 variants was evaluated for the specific antibody responses.

In this study, 18 amino acid changes sites were identified in PvAMA1 DI, 8 sites in DII and 4 sites in DIII compared to PvAMA1 (Sal-1 strain). The highest degree of polymorphism was found in DI, as previously observed in *P*. *vivax* isolates [24, 46]. In addition to being the most polymorphic domain [19, 47, 48] DI also contains the binding site to RON2, which acts in actin-myosin-associated moving junction formation. RON2 binds to a conserved portion of AMA1 [49] that is surrounded by highly polymorphic regions [50]. Therefore, DI is considered an important region for antibody response, while DII is considered to be the most immunogenic both in *P*. *falciparum* [51] and *P*. *vivax* [17].

B-cell epitopes were predicted on the protein structure by taking into account the importance of SNP localization to the immune response and selective pressure [52]. Notably, most polymorphic sites were found in regions of B-cell epitopes. Since polymorphisms are found predominantly on the exposed protein surface [52], amino acid changes at B-cell epitope sites may directly affect the antibody response because mutations may inhibit binding and hinder the generation of immunological memory.

Five mutations were detected at DI and two at DII, localized at B-cell epitopes in H5 or H16. Although the consequences of these mutations in each variant are not known, it is important to consider that changes in protein sequence can affect the immunorecognition of those epitopes.

AMA 1 is one of the *Plasmodium* antigens with the highest vaccine potential [53]. Studies have shown that vaccine formulations containing AMA1 are able to produce antibodies and induce protection in rodent and primate models [15, 16, 54, 55]. Numerous trials have already been conducted to show the effectiveness of this candidate in a falciparum malaria vaccine [53]. AMA1 is the most studied blood-stage vaccine candidate; however, only FMP2.1/AS02A has shown efficacy in clinical trials [56].

Immunization with only one allele may not protect against parasites expressing different AMA1 alleles [57, 58]. Plasma from humans exposed to *P*. *falciparum* were tested against different AMA1 alleles. Most individuals had not only a specific response to conserved regions but also raised specific antibodies against polymorphic regions of the antigen [58]. In addition, immunization of rabbits with the PfAMA1 containing an allele that is capable of blocking the growth of homologous parasites does not inhibit the heterologous parasite strain [57]. This fact suggests that these genetic differences are a result of immune pressure and modifications in alleles may impair recognition of B-cell epitopes by elicited antibodies. Therefore, it is important not only to characterize the genetic diversity of the antigen but also to identify its immunogenic potential to enable a vaccine strategy to include a multiple-allelic variant to obtain a strong and effective response.

In this instance, the immunoassay results showed that the variants of PvAMA1 (V5 and V16) are highly reactive. However, a greater naturally acquired immune response against PvAMA1V5 was found among different haplotypes in individuals from Manaus, including the isolate belonging to H16. Manaus is a low transmission region. Furthermore, the naturally acquired immune response was also analyzed in a population from gold mining areas, representing a high endemic area. The prevalence of antibodies against PvAMA1V5 is higher in Manaus compared to Itaituba. Since the haplotypes were identified in samples from Manaus, the immune response of individuals living in this region would be higher due to circulating target strains. On the other hand, H5 is probably not distributed with the same frequency in Itaituba. This possibility highlights the importance of vaccine formulations containing frequent haplotypes from different malaria endemic areas. The high immunogenicity of AMA1 for most individuals exposed to *P*. *falciparum* and *P*. *vivax* during natural malaria infection was also observed in earlier studies [59].

The immune response of infected individuals was also compared to noninfected individuals who had previous episodes of malaria. Interestingly, although there was no significantly higher prevalence of antibodies in either group, noninfected individuals had a higher IgG reactivity index compared to infected patients. Thus, a possible maturation of the immune system over time can be inferred.

In addition, individuals from Itaituba showed a statistically significant correlation between PvAMA1V5 reactivity index and age. Nevertheless, the correlation between age and PvAMA1V16 reactivity index was not statistically significant. This correlation has been previously described for several *Plasmodium* antigens [60–63] and suggests age-dependent immunity, since there is evidence that individuals living in malaria-endemic regions have increased immune responses according to the number of reinfections [59] and become clinically immune after multiple malaria episodes [64]. In the individuals from Manaus, no statistically significant correlation was found between age and reactivity index for any of the variants either due to sample size or to the region of residence. Itaituba is a gold mining region with a high number of cases and consequently has a greater exposure to the parasite compared to the individuals living in Manaus, where the incidence of malaria is lower.

For vivax malaria, there are few studies to date testing PvAMA1 as a vaccine candidate [65–67]. A vaccination regimen with PvAMA1 presented promising results, showing the ability to elicit long-lasting PvAMA1-specific antibody responses, as well as memory T cell responses, in mice [67]. This vaccine was also able to elicit invasion inhibitory antibodies against diverse *P*. *vivax* strains [65]. However, the most promising studies testing AMA1 formulations for both falciparum malaria in humans, demonstrating 64% efficacy against vaccine-like strains, and for vivax malaria in monkeys, did not provide significant overall protection and showed a probable limitation related to the extensive polymorphisms of AMA1. This property could induce antibodies that show limited cross-inhibition of parasites expressing other variants [56, 66].

Multi-allele vaccine inducing a broad inhibitory antibody response are aimed at an effective malaria vaccine. Therefore, we suggest that vaccine trials based on polymorphic antigens be designed considering strain frequencies at individual study sites. The high reactivity and the possible maintenance of the immune response over time indicate that PvAMA1V5 may be a promising component of a vaccine formulation.

## Supporting information

S1 Fig Dendrogram of phylogenetic relationships between haplotypes of *pvama1* in Manaus. (TIF)

S2 Fig Characterization of recombinant proteins PvAMA1V5 and PvAMA1V16. A) SDS-PAGE analysis of ~77kDa rPvAMA1V5 and PvAMAV16 stained with Comassie Brilliant Blue. B) Circular Dichroism spectra of PvAMA1 variants. Dotted line (PvAMA1V16) and solid line (PvAMA1V5). MM: Molecular marker PageRuler Prestained Protein Ladder, 10-180kDa (Thermo Scientific).
(TIF)

S3 Fig Correlation and paired comparison of naturally acquired immune responses to PvAMA1 variants from the Itaituba population. A) Paired PvAMA1V5 and PvAMA1V16 IgG responses comparison (Wilcoxon signed-rank test, *p* < 0.0001, n = 171). B) Spearman correlation coefficient (*rho*) of IgG antibodies against each PvAMA1 variant and t-test p-value (*p*). C) Prevalence of responders and non-responders towards PvAMA1V5 and PvAMA16.
(TIF)

S4 Fig Correlation between immune responses to PvAMA1 haplotypes in distinct groups from Itaituba. A) Spearman correlation coefficient (*rho*) between PvAMA1V5 and PvAMA1V16 IgG antibodies and t-test p-value (*p*) against each variant in acutely infected individuals. B) Prevalence of immune response towards PvAMA1 variants in infected individuals, C) *rho* between PvAMA1V5 and PvAMA1V16 IgG antibodies and t-test p-value (*p*) in noninfected individuals. D) Prevalence of immune response towards PvAMA1 variants in noninfected individuals.
(TIF)

S5 Fig Correlation between age and antibody response towards to PvAMA1 variants. A) Spearman Correlation coefficient (*rho*) between PvAMA1 variants reactivity indexes and age from full Itaituba sample. B) *rho* between PvAMA1 variants reactivity indexes and age for infected individuals, C) *rho* between PvAMA1 variants reactivity indexes and age for noninfected individuals. T-test p-value (*p*).
(TIF)

S6 Fig Multivariate correlation coefficient in infected and noninfected individuals from Itaituba. A) Correlation matrix of the infected group. B) Correlation matrix of the noninfected group. Positive correlations are represented with red, and negative correlations are represented with blue squares. Pearson correlation was applied to verify associations between the PvAMA1 variant reactivity index and hemoglobin, hematocrit, platelets and plasmatic cytokines (IFN-γ, IL6, IL10, IL2, IL4, TNF-α). p<0.05 was considered significant. *r* values are indicated in the figure.
(TIF)

S1 Table Predicted linear B-cell epitopes and mutation sites based on the PvAMA1 reference sequence comparing PvAMA1 haplotypes. Amino acids colored red indicate polymorphisms.
(DOCX)
