# Supplementary figures and images for: Plasmodium vivax AMA1: Implications of distinct haplotypes for immune response
Source: PLoS Negl Trop Dis. 2020 Jul 8;14(7):e0008471. doi: 10.1371/journal.pntd.0008471 (PMC7371208; doi:10.1371/journal.pntd.0008471)

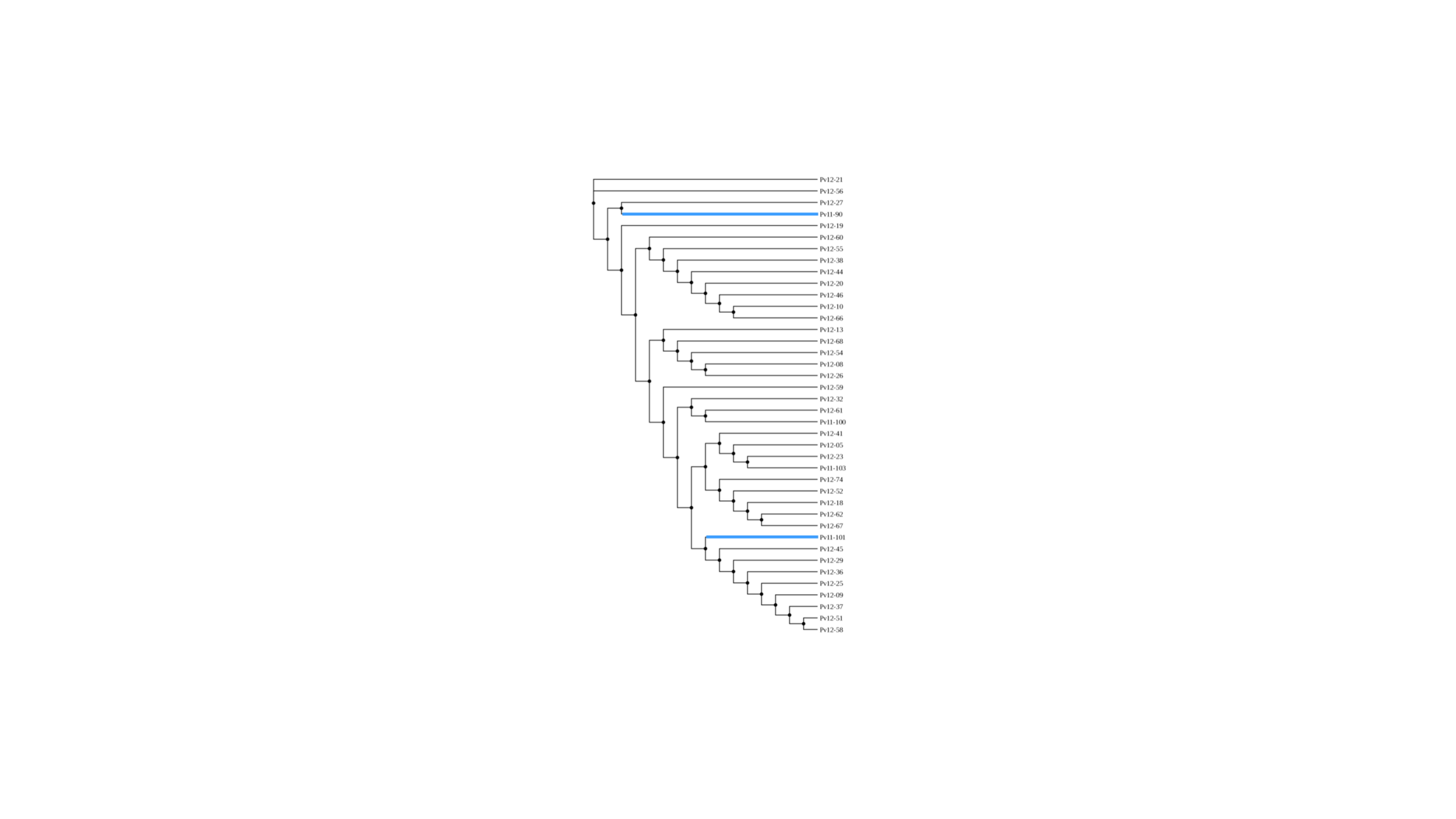

Supplement: S1 Fig — (TIF) [file pntd.0008471.s001.tif]

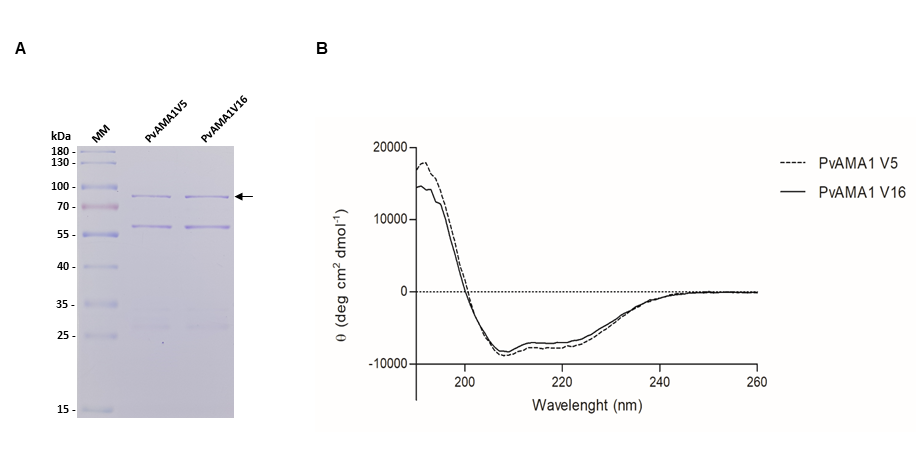

Supplement: S2 Fig — A) SDS-PAGE analysis of ~77kDa rPvAMA1V5 and PvAMAV16 stained with Comassie Brilliant Blue. B) Circular Dichroism spectra of PvAMA1 variants. Dotted line (PvAMA1V16) and solid line (PvAMA1V5). MM: Molecular marker PageRuler Prestained Protein Ladder, 10-180kDa (Thermo Scientific). (TIF) [file pntd.0008471.s002.tif]

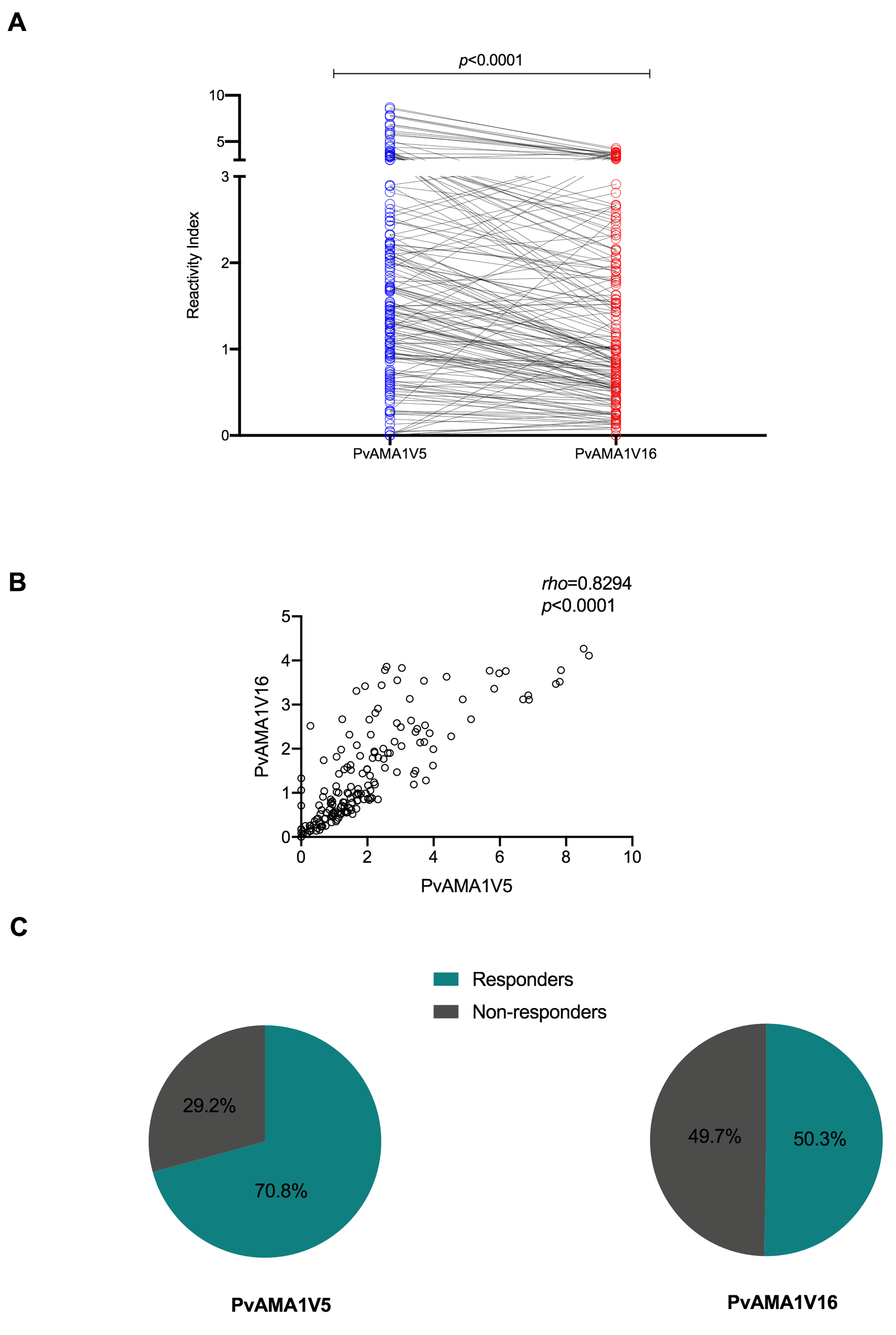

Supplement: S3 Fig — A) Paired PvAMA1V5 and PvAMA1V16 IgG responses comparison (Wilcoxon signed-rank test, p < 0.0001, n = 171). B) Spearman correlation coefficient (rho) of IgG antibodies against each PvAMA1 variant and t-test p-value (p). C) Prevalence of responders and non-responders towards PvAMA1V5 and PvAMA16. (TIF) [file pntd.0008471.s003.tif]

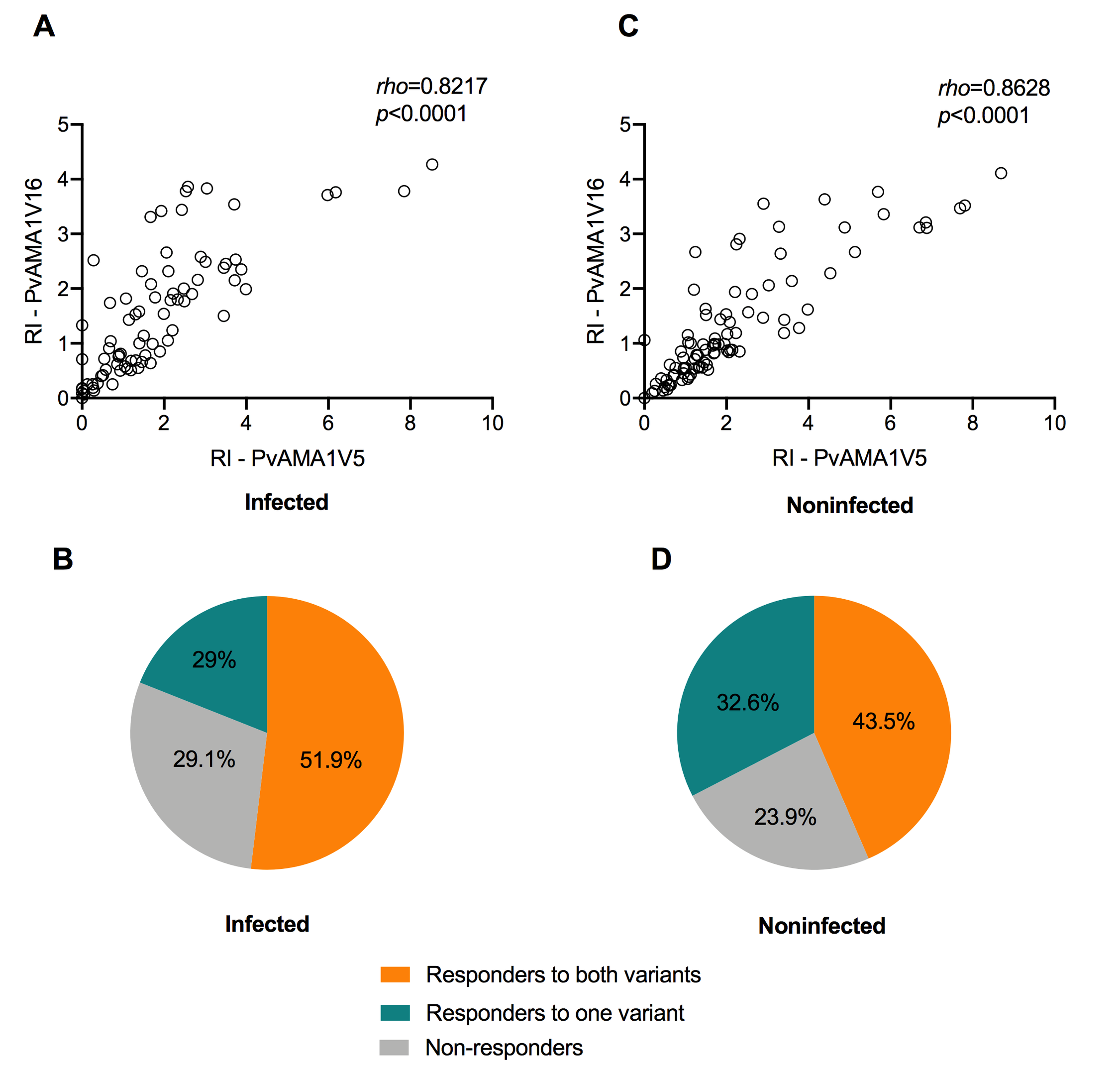

Supplement: S4 Fig — A) Spearman correlation coefficient (rho) between PvAMA1V5 and PvAMA1V16 IgG antibodies and t-test p-value (p) against each variant in acutely infected individuals. B) Prevalence of immune response towards PvAMA1 variants in infected individuals, C) rho between PvAMA1V5 and PvAMA1V16 IgG antibodies and t-test p-value (p) in noninfected individuals. D) Prevalence of immune response towards PvAMA1 variants in noninfected individuals. (TIF) [file pntd.0008471.s004.tif]

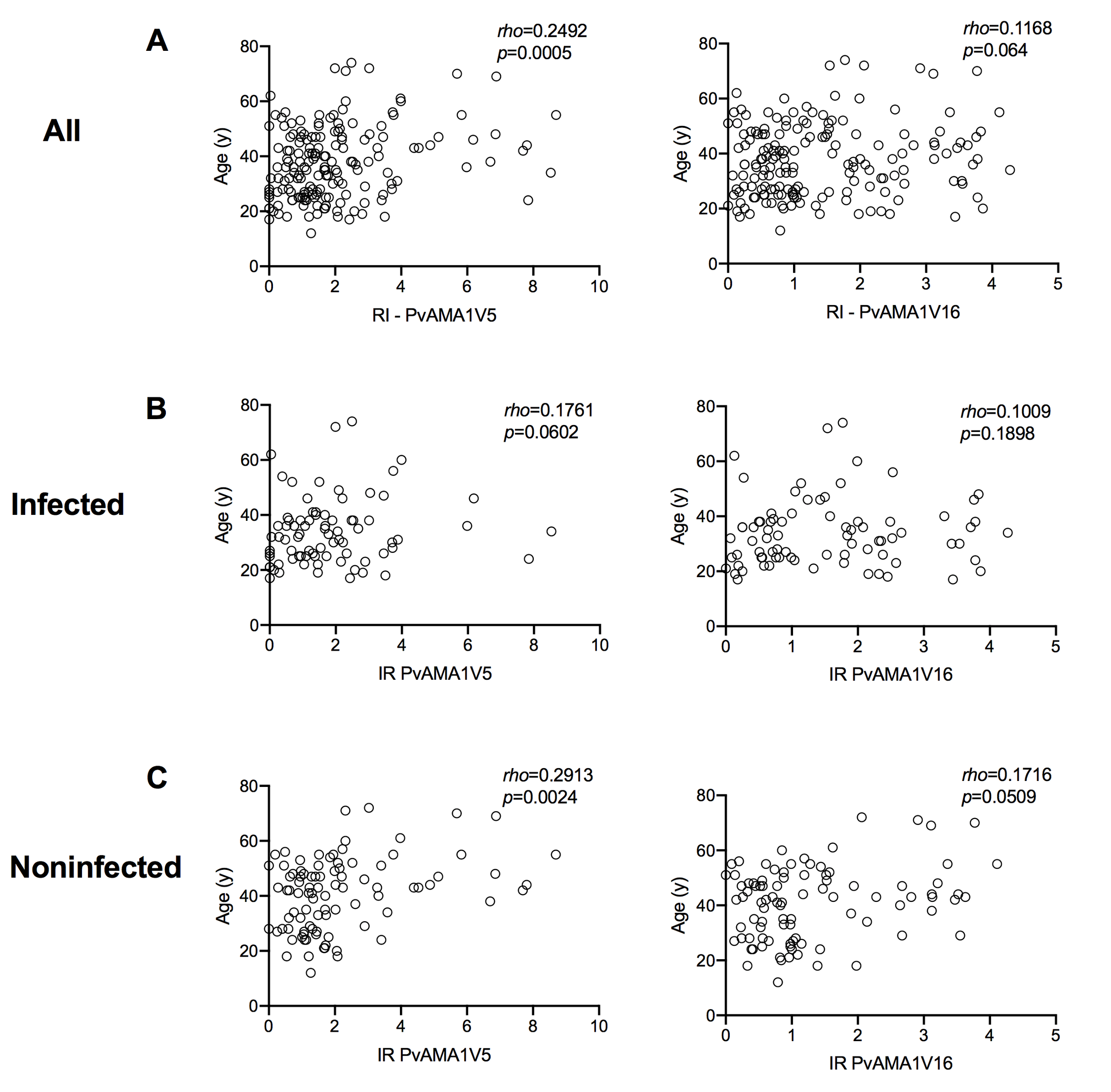

Supplement: S5 Fig — A) Spearman Correlation coefficient (rho) between PvAMA1 variants reactivity indexes and age from full Itaituba sample. B) rho between PvAMA1 variants reactivity indexes and age for infected individuals, C) rho between PvAMA1 variants reactivity indexes and age for noninfected individuals. T-test p-value (p). (TIF) [file pntd.0008471.s005.tif]

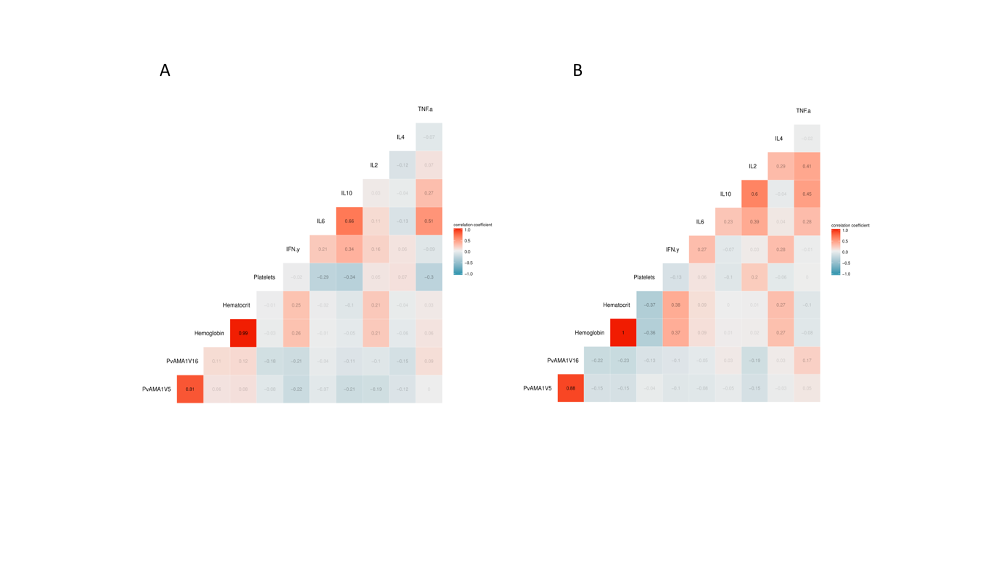

Supplement: S6 Fig — A) Correlation matrix of the infected group. B) Correlation matrix of the noninfected group. Positive correlations are represented with red, and negative correlations are represented with blue squares. Pearson correlation was applied to verify associations between the PvAMA1 variant reactivity index and hemoglobin, hematocrit, platelets and plasmatic cytokines (IFN-γ, IL6, IL10, IL2, IL4, TNF-α). p<0.05 was considered significant. r values are indicated in the figure. (TIF) [file pntd.0008471.s006.tif]
