# Supplementary material for: Plasmodium vivax AMA1: Implications of distinct haplotypes for immune response
Source: PLoS Negl Trop Dis. 2020 Jul 8;14(7):e0008471. doi: 10.1371/journal.pntd.0008471 (PMC7371208; doi:10.1371/journal.pntd.0008471)
Supplement: S1 Table — Amino acids colored red indicate polymorphisms. (DOCX) [file pntd.0008471.s007.docx]

| **Amino acid position** | **B-cell epitope** |
| --- | --- |
| 25-40 | QKPSRLTRSANNVLLE |
| 43-59 | PTVERSTRMSNPWKAFM |
| 63-83 | DIERTHSSGVRVDLGEDAEVE |
| 102-108 | VIENSDV |
| 117-121 | GDQKL |
| 130-136 | NANDHIS |
| 149-158 | DNVEMMKLND |
| 161-179 | LCRTHAASFVMAGDQNSSY |
| 189-216 | EKTCHMLYLSAQENMGPRYCSPDAQNRD |
| 236-265 | SKNVRNDWDKKCPRKNLGNAKFGLWVDGNC |
| 287-304 | FGASASDQPTQYEEEMTD |
| 331-339 | NSDNFKSKG |
| 344-350 | WANFDSV |
| 362-381 | TCLINDKNFIATTALSHPQE |
| 401-413 | KQSRNMNLYSVDG |
| 424-431 | SNDKESIK |
| 435-447 | EPERISNSTCNFY |
| 457-464 | EIKENNQV |
| 477-488 | GEEKSNKQMLLI |
| 512-521 | NNDKYDKMDQ |
| 531-535 | RKDEM |
| 545-558 | EDKRASHTTPVLME |
